# Nomograms for predicting Gleason upgrading in a contemporary Chinese cohort receiving radical prostatectomy after extended prostate biopsy: development and internal validation

**DOI:** 10.18632/oncotarget.7787

**Published:** 2016-02-29

**Authors:** Biming He, Rui Chen, Xu Gao, Shancheng Ren, Bo Yang, Jianguo Hou, Linhui Wang, Qing Yang, Tie Zhou, Lin Zhao, Chuanliang Xu, Yinghao Sun

**Affiliations:** ^1^ Department of Urology, Shanghai Changhai Hospital, Second Military Medical University, Shanghai, China

**Keywords:** prostate cancer, Gleason score upgrading, nomogram, prostate biopsy, prostatectomy

## Abstract

The current strategy for the histological assessment of prostate cancer (PCa) is mainly based on the Gleason score (GS). However, 30-40% of patients who undergo radical prostatectomy (RP) are misclassified at biopsy pathologically. Thus, we developed and validated nomograms for the prediction of Gleason score upgrading (GSU) in patients who underwent radical prostatectomy after extended prostate biopsy in a Chinese population. This retrospective study included a total of 411 patients who underwent radical prostatectomy at our institute after having prostate biopsies between 2011 and 2015. The final pathologic GS was upgraded in 151 (36.74%) of the cases in all patients and 92 (60.13%) cases in men with GS=6. In multivariate analyses, the primary biopsy GS, secondary biopsy GS and obesity were predictive of GSU in the patient cohort assessed. In patients with GS=6, the significant predictors of GSU included the body mass index (BMI), prostate-specific antigen density(PSAD) and percentage of positive cores. The area under the curve (AUC) of the prediction models was 0.753 for the entire patient population and 0.727 for the patients with GS=6. Both nomograms were well calibrated, and decision curve analysis demonstrated a high net benefit across a wide range of threshold probabilities. This study may be relevant for improved risk assessment and clinical decision-making in PCa patients.

## INTRODUCTION

The Gleason score (GS) is the most widely accepted system for the grading of prostate cancer (PCa) [1]. However, due to problems inherent with needle biopsy sampling, there is usually a difference between the GS at biopsy and the GS at radical prostatectomy (RP). According to the available reports, approximately 30-40% of patients who undergo RP are misclassified during the pathological review of the biopsy [2].

The decision-making process regarding the treatment options for patients with PCa, such as active surveillance, radical prostatectomy, brachytherapy or cryosurgery, is highly reliant on GS. Thus, precise determination of the GS at biopsyis of particular interest for clinical decision-making. For instance, GS upgrading (GSU) after RP is highly associated with a risk of extracapsular extension (ECE) and biochemical recurrence (BCR) [2]. In addition, it is also important that the presence of increasing quantities of Gleason pattern 4 results in an increased risk of biochemical disease recurrence, a need for adjuvant therapy, and cancer-specific mortality [3]. Importantly, there are substantial differences in the outcomes between patients with a GS of 3+4 and 4+3 or higher [4].

Although several nomograms for predicting GSU after RP have been reported [5–7], there is evidence that there is a high degree of racial variation in the upgrading and upstaging among patients of different races [8]. This is because the epidemiology and patient spectrum of PCa in China and other Asian countries with similar situations are quite different from those of Western populations [9, 10]. First, more Chinese patients are diagnosed with a higher grade GS; for example, patients diagnosed with a GS of ≥7 accounted for 80% of the total patients in a collaborative report in Asia [11]. We designed this study based on the Chinese population by introducing two nomograms: one for the overall patients and one for those patients with a GS=6. Second, a comparative study showed that the PCa prevalence on autopsy was similar between unscreened Caucasian and unscreened Asian Men; nevertheless, the rate of high-grade prostate cancer (HGPCa) was higher in unscreened Asian men than in unscreened Caucasian men, even after adjusting for age and prostate weight [12], suggesting differences in this grading system between the two populations. Third, reports from Korea and Japan have illustrated that Western population-derived prediction models perform poorly in Asian populations [13, 14]. Pilot studies have been performed in Asian populations [13, 15–17]; however, the sample size and factors involved were limited, and the nomogram derived from these studies lacked validation.

Thus, we performed this retrospective study to better predict GSU in a contemporary Chinese cohort of patients who underwent transrectal ultrasound (TRUS)-guided biopsy and subsequent RP. Nomograms predicting significant GSU and any upgrading were established and internally validated.

## RESULTS

### Patient characteristics

The clinical and pathologic characteristics of all involved patients and those with a GS=6 are shown in Table 1. The final pathologic GS was upgraded in 151 (36.74%) cases in overall patient population and in 92 (60.13%) cases among men with a GS=6. A total of 61 (39.87%) men with a GS=6 and 168 (40.88%) of the overall patients had the same GS at biopsy and RP. Nevertheless, 92 (22.38%) patients in the overall patient showed downgrading of the GS from biopsy to RP (Table 2).

**Table 1:** Clinicobiologic and pathologic characteristics of involved patients

Variable	Overall cohort	GS=6 Cohort
Age, Mean (SD), yr:	67.12 (7.24)	66.06 (7.25)
BMI, Mean (SD)	24.07 (2.78)	23.84 (2.95)
BMI≥24	215 (52.31)	75 (49.02)
BMI≥28	26 (6.33)	10 (6.54)
PSA, Median (IQR), ng/ml	10.84 (12.85)	9.7 (9.16)
PV, Median (IQR), ml:	32.76 (22.23)	36.4 (27.04)
PSAD, Median (IQR), ng/ml per gram	0.33 (0.44)	0.29 (0.33)
clinical stage		
t1c	80 (19.46)	37 (24.18)
t2a	65 (15.82)	25 (16.34)
t2b	166 (40.39)	67 (43.79)
t2c	67 (16.30)	21 (13.73)
t3	22 (5.35)	1 (0.65)
t4	11 (2.68)	2 (1.31)
Biopsy specimens features:		
Biopsy cores, Median (IQR)	12 (1.00)	12 (2.00)
Postive cores, Median (IQR)	4 (4.00)	2 (3.00)
% of positive cores, Median (IQR), %	31 (33.33)	20 (23.33)
Biopsy gleason score, No (%)		
6	153 (37.23)	153 (100)
3+4	84 (20.44)	-
4+3	52 (12.65)	-
8	70 (17.03)	-
9	41 (9.98)	-
10	11 (2.68)	-
RP specimens features:		
RP Gleason score, No (%)		
6	81 (19.71)	61 (39.87)
3+4	158 (38.44)	65 (42.48)
4+3	60 (14.60)	17 (11.11)
8	51 (12.41)	6 (3.92)
9	55 (13.38)	4 (2.61)
10	6 (1.46)	-
PSM, No (%)	103 (25.06)	27 (17.65)
SVI, No (%)	48 (11.68)	4 (2.61)
EMI, No (%)	89 (21.65)	19 (12.42)
LMP, No (%)	11 (2.68)	-
Nerve, No (%)	117 (28.47)	25 (16.34)

PV=prostate volume; PSM=positive surgerical margin; SVI=seminal vesicle invasion; EMI=extraprostic invasion; LMP=lymph node positive;

**Table 2:** Paired comparison of biospy and radical prostatectomy Gleason scores in patients with prostate cancer (n=411)

Biopsy Gleason Score	RP Gleason Score						Total
	6	3+4	4+3	8	9	10	
6	61 (14.84%)	65 (15.82%)	17 (4.14%)	6 (1.46%)	4 (0.97%)	-	153 (37.23%)
3+4	10 (2.43%)	51 (12.41%)	13 (3.16%)	6 (1.46%)	4 (0.97%)	-	84 (20.44%)
4+3	3 (0.73%)	21 (5.11%)	9 (2.19%)	14 (3.41%)	5 (1.21%)	-	52 (12.65%)
8	4 (0.97%)	15 (3.65%)	14 (3.41%)	21 (5.11%)	13 (3.16%)	3 (0.73%)	70 (17.03%)
9	2 (0.49%)	6 (1.46%)	6 (1.46%)	2 (0.49)	24 (5.84%)	1 (0.24%)	41 (9.98%)
10	1 (0.24%)	-	1 (0.24%)	2 (0.49)	5 (1.21%)	2 (0.49)	11 (2.68%)
Total	81 (19.71%)	158 (38.44%)	60 (14.60%)	51 (12.41%)	55 (13.38%)	6 (1.46%)	411 (100%)

RP=radical prostatectomy

### Predictors of GSU in the overall patient population

**Figure 1:**
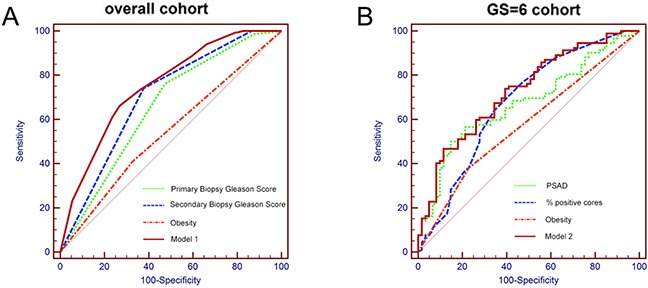
Receiver operating characteristic (ROC) curves of the prediction models and single predictors in predicting GSU in **A.** all men and **B.** men with GS=6.

In univariate logistic regression analyses of potential preoperative predictors of GSU in the overall patient pool, primary biopsy GS (P<0.001) and secondary biopsy GS (P<0.001 in patients with GS=4) were statistically significant predictors of GSU (Table 3). The only informative predictors (P<0.001) in the overall patient group were the primary and secondary biopsy GS values (AUC0.66 and 0.70, respectively),both of which were negatively correlated with GSU. Although obesity was correlated with GSU at the borderline significance P value of 0.089 in the overall cohort, obese patients were estimated to have a 2.6-fold higher risk of GSU than non-obese patients. Thus, we tested the performance of this variable in the multivariate prediction model. In the multivariate logistic regression analysis, the predictors were obtained using the backward elimination selection procedure, including the primary biopsy GS=4, primary GS=5; secondary biopsy GS=4, secondly GS=5; and obesity, with an OR of 0.53 (95%CI, 0.32 to 0.87), 0.14 (95%CI, 0.03 to 0.62); 0.30 (95%CI, 0.19 to 0.48), 0.00 (95%CI, 0.00 to 0.00); and 1.72 (95%CI, 1.08 to 2.74), respectively. The AUC of the prediction model reached 0.753 (95%CI, 0.706 to 0.800) (Table 4, Figure 1).

**Table 3:** The results of the univariate logistic regression model of predictors for GSU and GSU from GS=6

Predictors	Predicting any GSU				AUC	Predicting GSU form GS=6				AUC
	OR	95%CI	P	beta-coefficent		OR	95%CI	P	beta-coefficent	
Primary GS					0.66	-				
3	1.00 (Reference)									
4	0.35	0.22-0.02	<0.001	−1.06						
5	0.07	0.02-0.29	<0.001	−2.71						
Secondary GS					0.70	-				
3	1.00 (Reference)									
4	0.27	0.17-0.42	<0.001	−1.31						
5	0	0.00-0.00	0.997	−21.34						
non-obesity (BMI<28)	1.00 (Reference)									
Obesity (BMI>28)	1.43	0.95-2.17	0.089	0.36	0.54	2.06	0.99-4.28	0.052	0.72	0.58
Age (ys)					0.51					0.53
<60	1.00 (Reference)					1.00 (Reference)				
60-70	1.23	0.68-2.22	0.683	0.21		1.60	0.67-3.80	0.287	0.47	
>70	1.00	0.54-1.86	0.991	−0.64		1.43	0.56-3.61	0.456	0.35	
DRE	0.91	0.58-1.43	0.682	−0.10	0.51	0.96	0.43-2.13	0.922	−0.40	
BMI	0.99	0.97-1.01	0.534	−0.01	0.55	1.08	0.97-1.20	0.170	0.08	0.57
PSA level (ng/ml)	1.00	0.99-1.02	0.657	1.00	0.54	1.05	1.01-1.09	0.013	0.05	0.65
PSAD	1.05	0.76-1.46	0.765	0.76	0.55	11.11	2.50-49.38	0.002	2.41	0.67
N of cores taken						0.75	0.36-1.55	0.432	−0.29	0.52
<12	1.00 (Reference)					1.00 (Reference)				
≥12	1.31	0.84-2.06	0.238	0.27		1.31	0.84-2.06	0.238	0.27	
N positive cores (n)	1.02	0.96-1.09	0.494	0.02		1.24	1.05-1.47	0.012	0.22	0.66
% positive cores	1.21	0.58-2.53	0.606	0.19		11.38	1.63-79.70	0.014	2.43	0.68
Clinical stage					0.54					0.56
T1c	1.00 (Reference)					1.00 (Reference)				
T2a	1.20	0.62-2.38	0.598	0.18		2.43	0.75-7.96	0.141	0.89	
T2b	0.89	0.52-1.55	0.692	−0.11		0.71	0.31-1.61	0.407	−0.35	
T2c	0.83	0.42-1.62	0.580	−0.19		0.67	0.23-1.98	0.468	−0.40	
T3	0.59	0.24-1.44	0.249	−0.52		1.22	0.01-14.70	0.877	0.20	
PV (ml)					0.50					0.56
<30	1.00 (Reference)					1.00 (Reference)				
30-45	0.65	0.40-1.06	0.085	0.40		0.60	0.26-1.36	0.222	−0.51	
>45	1.02	0.63-1.66	0.938	0.02		0.70	0.32-1.52	0.361	−0.36	
Surgical technique					0.51					0.53
ORP	1.00 (Reference)					1.00 (Reference)				
LRP	0.90	0.52-1.55	0.702	−0.11		0.69	0.29-1.64	0.401	−0.37	
RALP	1.08	0.67-1.76	0.750	0.08		1.07	0.50-2.30	0.867	0.07	

P <0.05 is statistically significant.

**Table 4:** The results of the multiple logistic regression model of predictors for GSU and GSU from GS=6

Predictors	Predicting any GSU				Predictors	Predicting GSU form GS=6			
	OR	95%CI	P	beta-coefficent		OR	95%CI	P	beta-coefficent
Primary GS					Obesity (BMI>28)	2.02	0.93-4.40	0.077	0.70
3	1.00 (Reference)								
4	0.53	0.32-0.87	0.012	−0.64					
5	0.14	0.03-0.62	0.010	−1.98	PSAD	9.66	2.16-43.17	0.003	2.27
Secondary GS									
3	1.00 (Reference)								
4	0.30	0.19-0.48	0.000	−1.21	% positive cores	10.96	1.54-78.09	0.017	2.39
5	0.00	0.00-0.00	0.998	−20.66					
Obesity (BMI>28)	1.72	1.08-2.74	0.023	0.54	Constant			0.003	−1.18
AUC	0.753 (0.706-0.800)				AUC	0.727 (0.647-0.808)			

### Predictors of GSU in men with GS=6

Univariate logistic regression analyses indicated that the preoperative variables significantly associated with GSU included PSA (P=0.013), PSAD (P=0.002), the number of positive cores (P=0.012) and the percentage of positive cores(P=0.014), which showed ORs of 1.05 (95%CI, 1.01 to 1.09), 11.11 (95%CI, 2.50 to 49.38), 1.24 (95%CI, 1.05 to 1.47) and 11.38(95%CI, 1.63 to 79.70), respectively. The percentage of positive cores represented the highest AUC (0.68) in predicting the GSU and was followed by the PSAD (0.67), the number of positive cores (0.66) and the PSA (0.65). In addition, obesity (defined as a BMI>28 in Chinese individuals, according to the official standards) was associated with GSU at a borderline significance level (P=0.052), with an AUC of 0.58. In the multivariate analyses, the predictors of GSU included BMI (P=0.077), PSAD (P=0.003) and the percentage of positive cores (P=0.017), with a relative increase of 2.02 (95%CI, 0.93 to 4.40), 9.66 (95%CI, 2.16 to 43.17) and 10.96 (95%CI 1.54 to 78.09), respectively (Table 4). The accuracy of this prediction model was relatively high, with an AUC of 0.727 (95%CI, 0.647 to 0.808) (Table 4, Figure 1).

### Calibration curve analyses

**Figure 2:**
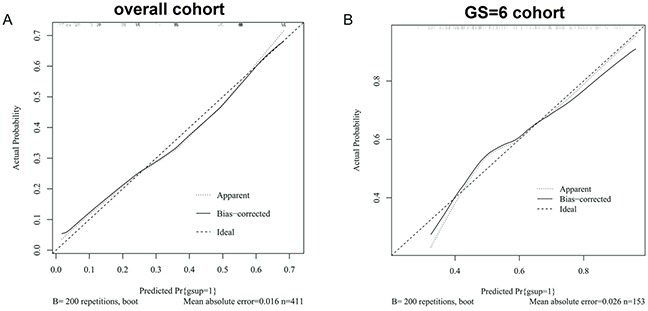
Calibration curves of prediction models in **A.** the overall patient population and **B.** the cohort of GSU from GS=6 across all probabilities of GSU.

In addition to the AUC, calibration is an important indicator of nomogram performance. Thus, calibration analysis was applied to measure how far the predictions were from the actual outcomes. The bias-corrected calibration plots showed only a limited departure from the ideal predictions. The mean absolute error was 1.6% in the overall patient group and 2.6% in the GS6 cohort (Figure 2).

### Decision curve analyses

**Figure 3:**
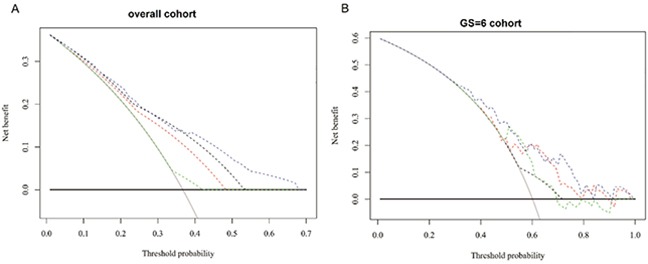
Decision analysis demonstrated ahigh net benefit across a wide range of threshold probabilities in **A.** the overall patient population (model 1) and **B.** the cohort of men with GS=6(model 2). (A): Black: biopsy none; Grey: biopsy all; Dotted black: the primary GSat biopsy; Dotted red: the secondary GSat biopsy; Dotted green: obesity; Dotted Blue: Model 1. (B): Black: biopsy none; Grey: biopsy all; Dotted black: obesity; Dotted red:PSAD;Dotted green: percentage of positive cores; Dotted Blue: Model 2.

In the decision analyses, the net benefit was higher for the two models at the highest threshold compared with any single predictor of GSU in the overall patient group and in those with a GS=6 (Figure 3). Higher positive net benefits were observed in the range of most threshold probabilities from 0.4 to 0.7 in any GSU and from over 0.3 to 0.8 in the GSU for patients with a GS=6, suggesting a benefit in men with a probability in these ranges. In the overall patient population, the primary biopsy GS showed the highest performance, followed by the secondary biopsy GS and obesity. In men with a GS=6, Model 2 showed better performance in most thresholds, from 0.3 to 0.8.

## DISCUSSION

**Figure 4:**
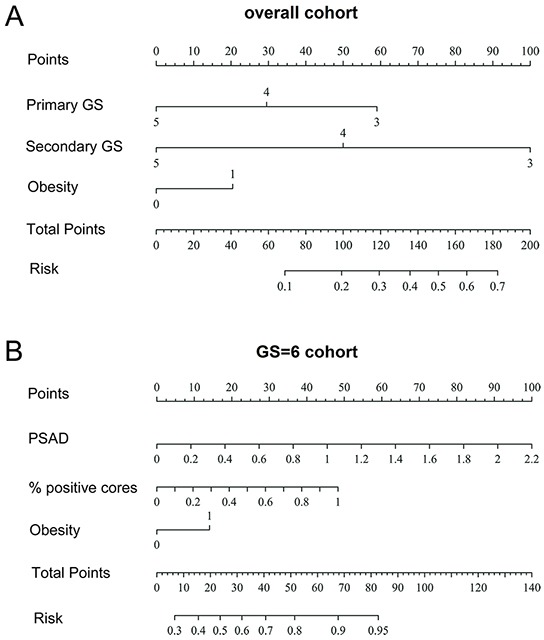
The nomogram of GSU prediction in **A.** the overall patient population and **B.** the patients with GS=6. Instructions for physicians: To obtain the nomogram-predicted probability of GSU, locate the patient values at each axis. Draw a vertical line to the “Points” axis to determine how many points are attributed for each variable value. Sum the points for all variables. Locate the sum on the “Total Points” line to assess the individual probability of cancer on prostate biopsy on the “Risk” line. Primary GS: the primary GSat biopsy; Secondary GS: the secondary GSat biopsy; PSAD: prostate-specific antigen density; % positive cores: percentage of positive cores.

In this study, we found that the primary biopsy GS and secondary biopsy GS values were significant predictors of GSU in all patients undergoing RP, while the PSA, PSAD, number of positive biopsy cores, percentage of positive cores, and obesity were predictors of GSU in patients with GS=6. Prediction models including the primary biopsy GS, secondary biopsy GS and obesity were constructed for the overall patient population, while the prediction model in patients with GS=6 included the PSAD, percentage of positive cores and obesity (Figure 4). Both models were of a relatively high accuracy and were well fitted in the calibration analysis; clinical decision curve analysis also confirmed their effectiveness.

Several models predicting GSU after RP have been constructed, mostly in Western patient populations. For instance, the primary biopsy GS, secondary biopsy GS, preoperative PSA and clinical stage were identified as predictors of GSU in a study by Chun FK et al. [5]. In particular, patients with a primary biopsy GS ≤3 or secondary biopsy GS≤3 were likely to have GSU, and a higher preoperative PSA level was correlated with an increased likelihood of GSU.

However, the PSA level was not identified as a significant predictor of GSU in this study. We suggest that this may be due to the differences in the PSA distribution between the two studies. There are 25% of patients had a PSA level over 19.3 ng/ml in this study, while only a small portion of patients (7.6%) had a PSA level over 20 ng/ml in the study conducted by Chun FK et al. This fact further illustrates the necessity of establishing new prediction models in the Chinese population, which appears to be quite different from the Western population.

Furthermore, a study in a Japanese population also confirmed that men with a primary biopsy GS of 4-5 show a much lower likelihood of GSU [17]. The primary biopsy GS was found to be more accurate than the secondary biopsy GS in the Japanese study. However, the predictive accuracy of secondary biopsy GS was higher than that of the primary biopsy GS both in this study and the study conducted by Chun et al. We suggest that differences in the biopsy GS may account for this difference.

Studies focused on patients with GS=6, especially those with the potential to meet the inclusion criteria for active surveillance or other conservative treatment options such as brachy therapy and cryosurgery (CSAP), are therefore important. Truong M et al. [6] developed a prediction model in a total of 1,961 patients who underwent RP with a GS=6. PSAD, obesity (BMI>30), and maximum core involvement were correlated with the GSU. Similarly, PSAD was considered to be a predictor of GSU in several previous studies [19–22]. Tilki D et al. [21] found that PSAD was a strong predictor of GSU, and our results confirm this finding. Nevertheless, we found that neither the PSA nor the prostate volume was significantly associated with GSU. We thus predict that the effect of these two predictors may be enhanced when they are combined.

Previous reports have suggested that the prostate volume is a significant predictor of GSU [17, 19]. Data from this study suggest that patients with a median prostate size (30-45 ml) may have a lower risk of GSU than patients with smaller prostates (<30 ml) in the overall patient population (OR=0.65, 95%CI, 0.40 to 1.06, P=0.085) and in patients with GS=6 (OR=0.60, 95%CI, 0.26 to 1.36, P=0.222). However, the trend was reversed when the prostate volume continued to increase (30-45 ml vs. > 45 ml). We thus predict that there are confounding factors in the relationship between prostate volume and GSU, although the limited sample size of this study makes it difficult to determine the exact influence of this variable.

This study supports the recent findings that BMI or obesity may have a significant correlation with GSU, as patients with a higher BMI show a higher likelihood of GSU [23]. Although the BMI was not significantly associated with GSU (P=0.534 in overall patients and P=0.170 in patients with GS=6), we found that obese patients showed a higher likelihood of GSU. Although there are racial differences in BMI between populations, this study suggests that we should be cautious about GSU in men with a higher BMI in the Chinese population.

Age has been considered an important predictor of GSU in Western studies [8,18,21]. Nevertheless, we found that age was not a significant predictor in this study. Such variation may be due to the limited sample size and different composition of the included patients. The mean age of this Chinese cohort was 67 years, which was much higher than that of previous Western reports (58.6-64.3 years) [6, 7, 19, 21]. Other factors, such as the tumor volume at biopsy and the prostate weight at RP, have also been shown to be associated with GSU in previous reports [6, 19]; nevertheless, these factors were not included in this study.

On the whole, there was a relatively large difference between this Chinese nomogram and the Western nomogram in predicting GSU in the overall patient population. However, the nomograms predicting GSU in patients with GS=6 were similar between the two populations, which may be due to the differences in the characteristics of PCa patients in the two populations. Currently, most PCa patients are diagnosed at an earlier stage and with a lower GS(a high proportion of patients with GS=6) in Western countries due to the implementation of PSA-based screening. In China, however, a high proportion of patients are diagnosed with a GS of 3+4 or 4+3 and even higher. Thus, we need to develop prediction models in these special situations, as the prediction of GSU in these patients would influence decision-making regarding RP and other treatments.

This study presented the first prediction tool of GSU for Chinese patients with a GS=6 who underwent RP after biopsy. The developed nomogram was based on more clinical predictors and a larger sample size than previous studies [16] for overall patients. These strengths will enhance its performance in future clinical practice.

Nevertheless, there were some drawbacks to this study. First, this was a retrospective study and thus suffers the limitations associated with this type of study. Furthermore, some predictors were not involved in the analyses, such as the percent of tumor in each core and the tumor size. Although the prostate volume of most patients was assessed by TRUS, there were a few patients who without these data, and the information regarding prostate volume for these patients instead came from the RP specimens. Lastly, the sample size of this study was limited, and only single-center data were involved. As the Chinese Prostate Cancer Consortium was established to facilitate multi-center studies, a PCa database based on browser/sever schema was created [24]. Importantly, the external validation of these nomograms in a multi-center population is scheduled.

## MATERIALS AND METHODS

### Patient population

From 2011 to 2015, a total of 642 consecutive patients who underwent radical prostatectomy at our institute after TRUS-guided prostate biopsies were retrospectively involved in this study. Of these, 231 (35.98%) were excluded because of missing information (n=129) or inadequate prostate sampling (<10 cores, n=95) or sarcoma (n=7). Further analysis targeted the remaining 411 patients. The clinical and pathologic characteristics of the involved patients, including age, body mass index (BMI), preoperative PSA, prostate volume measured by TRUS, prostate-specific antigen density(PSAD), clinical stage, biopsy and RP specimen features, are shown in Table 1. GSU was defined as a biopsy GS changing from 6 to 7 or changing from 7 to a higher GS, as well as a GS changing from 3+4 to 4+3. GSU in patients with GS=6 was defined as changes in the GS from 6 to 7 or more.

Both biopsy and prostatectomy specimens were evaluated by the same uropathology group. Nine priori-defined preoperative risk factors, including patient age, BMI, obesity, PSA, prostate volume, PSAD, clinical stage, number of positive cores, and percentage of positive cores, were assessed for their ability to predict GSU in patients with GS=6. Another two factors, the primary and secondary biopsy GS values, were added to the 9 factors for the assessment of GSU in all cases. We were unable to assess the percentage of cancer tissue involved in each biopsy core due to incomplete data.

### Statistical analyses

Statistical analysis was performed in the overall patient population and the patients with GS=6. First, univariate logistic regression was performed to investigate the association of clinical and pathological variables with GSU and GSU from GS=6. Second, variables with P<0.1 in the univariate analysis were further tested for in multivariable logistic regression analysis to identify independent predictors of GSU or GSU from GS=6. We chose to use P<0.1 as the criterion because we intended to expand the inclusion of variables. Third, receiver-operating characteristic (ROC) curve analysis was applied to calculate the area under the curve (AUC) of the prediction models. Fourth, calibration curves were constructed to assess the agreement between the actual rate of GSU and the predicted probabilities of GSU by these two models. The bias-corrected calibrated values were generated from internal validation based on 200 bootstrap resamples. The ideal curve was characterized with an intercept close to 0 and a slope close to 1. Finally, the decision curve analysis described by Vickers et al. [18] was performed to assess the clinical utility of these two models and the single predictors by quantifying the net benefits at a spectrum of threshold probabilities. All of the P values were two-sided, and P<0.05 was considered to be statistically significant. ROC curve analysis was performed using MedCalc v.10.4.7.0 (MedCalc Software bvba, Mariakerke, Belgium), and other analyses were performed with R version 3.1.3 (R foundation for Statistical Computing, Vienna, Austria).

## CONCLUSION

Obesity and primary and secondary biopsy GS values were identified as predictors of GSU in the overall patient population. Obesity, PSAD and the percentage of positive cores served as predictors of GSU in patients with a GS=6. Nomograms for predicting GSU were established in Chinese PCa patients, with a relatively high accuracy in internal validation. This study may therefore be of relevance in the risk assessment and clinical decision-making for PCa patients.
